# Epigenetic regulation of CpG promoter methylation in invasive prostate cancer cells

**DOI:** 10.1186/1476-4598-9-267

**Published:** 2010-10-07

**Authors:** Lesley A Mathews, Elaine M Hurt, Xiaohu Zhang, William L Farrar

**Affiliations:** 1 Cancer Stem Cell Section, Laboratory of Cancer Prevention, Center for Cancer Research, National Cancer Institute at Frederick, Frederick, MD 21702, USA; 2 Cancer Stem Cell Section, Laboratory of Cancer Prevention, SAIC-Frederick Inc., National Cancer Institute at Frederick, Frederick, MD 21702, USA

## Abstract

**Background:**

Recently, much attention has been focused on gaining a better understanding of the different populations of cells within a tumor and their contribution to cancer progression. One of the most commonly used methods to isolate a more aggressive sub-population of cells utilizes cell sorting based on expression of certain cell adhesion molecules. A recently established method we developed is to isolate these more aggressive cells based on their properties of increased invasive ability. These more invasive cells have been previously characterized as tumor initiating cells (TICs) that have a stem-like genomic signature and express a number of stem cell genes including *Oct3/4 *and *Nanog *and are more tumorigenic compared to their 'non-invasive' counterpart. They also have a profile reminiscent of cells undergoing a classic pattern of epithelial to mesenchymal transition or EMT. Using this model of invasion, we sought to investigate which genes are under epigenetic control in this rare population of cells. Epigenetic modifications, specifically DNA methylation, are key events regulating the process of normal human development. To determine the specific methylation pattern in these invasive prostate cells, and if any developmental genes were being differentially regulated, we analyzed differences in global CpG promoter methylation.

**Results:**

Differentially methylated genes were determined and select genes were chosen for additional analyses. The non-receptor tyrosine kinase BMX and transcription factor SOX1 were found to play a significant role in invasion. Ingenuity pathway analysis revealed the methylated gene list frequently displayed genes from the IL-6/STAT3 pathway. Cells which have decreased levels of the targets BMX and SOX1 also display loss of STAT3 activity. Finally, using Oncomine, it was determined that more aggressive metastatic prostate cancers in humans also have higher levels of both *Stat3 *and *Sox1*.

**Conclusions:**

Using this method we can begin to understand which genes are epigenetically regulated in the invasive population compared to the bulk tumor cells. These aggressive sub-populations of cells may be linked to the cancer stem cell hypothesis, making their patterns of epigenetic regulation very attractive for biomarker analysis.

## Background

Cancer is defined as uncontrolled cell growth resulting from genetic mutations or exposure to environmental carcinogens that alter normal regulation. If the cancer is aggressive in nature, invasion of local tissues near the primary tumor site as well as distant metastasis can occur. Current treatment regimens almost always involve a form of surgery to remove the primary tumor and systemic chemotherapy with localized radiation. However, aggressive cells can remain in the body and evade treatment with these conventional therapies. Additionally, it has been well documented that only a small fraction of epithelial tumor cells have the ability to form colonies *in vitro *or to initiate a new tumor upon injection into a host *in vivo *[1-6]. In order to study the epigenetic regulation of these aggressive cells, we chose to study an invasive population of prostate cancer cells. We and others have developed a novel method for the isolation of these cells from bulk tumor cell populations using Matrigel [7,8]. These cells have a stem-like phenotype [7] and exist within both established cell lines (LNCaP and DU145) and in cells isolated from primary prostate cancer tissue (PCSC1-3). The invasive cells have been characterized as undergoing an epithelial to mesenchymal transition (EMT) during the process of invasion, and are also highly tumorigenic when injected into mice [7]. They demonstrate increases in the stem cell regulators *CD44, CD133, Bmi1, Nanog*, and *Sonic hedgehog *(*Shh*), as well as increased expression in mesenchymal markers such as *Vimentin *and *Tgfβ-1*, and a decrease in the epithelial marker *E-cadherin *(*CDH1*). Over the last few years this hypothesis of EMT and cancer progression has been widely supported in models of not only prostate cancer, but also within the breast, colon, lung and pancreas [9-16]. The idea that the same cells which are undergoing the EMT may also be a population of cells called cancer stem cells or CSCs is a relativity new concept.

It is becoming more evident that CSCs are not governed by the same type of genetic regulation as normal stem cells, and arguably in solid tumors may be an epithelial cell that has up-regulated pathways that have been previously observed in true stem cells. In order to determine the epigenetic profile of these invasive prostate cancer cells, we isolated DNA and performed a very sensitive MeDIP (methylated DNA immunoprecipitation) assay coupled with Agilent's 244 K Human Promoter Tiling Arrays. This allowed for an in-depth analysis of the methylation status within promoter elements, upstream as well as down, in these cells. Differences between the invaded (more stem-like) and non-invaded cells, as well as the bulk tumor cell line (parental cells) were compared. In our analysis, the LNCaP and DU145 cell lines were used, as well as confirmation analysis in two primary prostate cancer cell lines (PCSC1 and PCSC2).

A unique set of genes were found to be expressed in the invasive cells, yet methylated in the non-invasive cells and parental cell lines. This included genes involved in embryonic and tissue/organ development, and specifically in neurogenesis including bone marrow X kinase (*Bmx*), Iroquois homeobox 3 (*Irx3*), Sine oculis homeobox homolog 1 (*Six1*) and Sex determining region-Y-box 1 (*Sox1*). Using the available online expression databases in Oncomine, it was determined that *Sox1 *plays a significant role in prostate cancer progression and metastasis. Furthermore, Ingenuity pathway analysis determined that the set of differentially methylated genes are involved in cellular functions such as cell-to-cell interaction and cell morphology, as well as development of the hematological system and cancer. The most intriguing data identified many of the methylated targets as members of the IL-6/STAT3 signaling pathway. Further investigation demonstrated that *Stat3 *was increased in these invasive cells, and cells infected with an shRNA against either BMX or SOX1 resulted in decreased levels of activated STAT3. However, only the differentially methylated *Sox1 *directly interacts with STAT3. Thus, in our model SOX1 plays a critical role in regulating invasive prostate cancer cells. These aggressive sub-populations of cells may be linked to the cancer stem cell hypothesis, making their patterns of epigenetic regulation very attractive for biomarker analysis.

## Materials and methods

### Cell Lines and Reagents

LNCaP and DU145 human prostate cancer cell lines were obtained from ATCC and cultured accordingly (Manassas, VA). Primary human prostate cancer cells (PCSC1-2) [7] were acquired from Celprogen (San Pedro, CA) and maintained as recommended using specific coated culture plates and defined media. Human bone marrow derived mesenchymal stem cells (hMSCs) were obtained from Lonza (Gaithersburg, MD) and maintained using their recommended conditions. The cultures were maintained in 5% CO_2 _air at 37°C. Human serum was obtained from Gemini Bioproducts (West Sacramento, CA). The following inhibitors were also used: Anti-human IL-6 antibody (R&D Systems, Minneapolis, MN), PI3K inhibitor LY294002 (Cell Signaling, Danvers, MA), Tec Kinase inhibitor LFM-A13 (Tocris, Ellisville, MO), MEK inhibitor PD98059 (Gibco, Carlsbad, CA), JAK inhibitor AG490 (Gibco), and STAT3 inhibitor Stattic (Sigma Aldrich, St. Louis, MO).

### Matrigel Invasion Assay

Matrigel-coated 24-well inserts (8 μM pore size) and non-coated control inserts purchased from BD Biosciences (Palo Alto, CA) were used according to manufacturer's instructions. A range of 20,000-100,000 cells were seeded for the invasion (higher for less invasive LNCaP cells). Cells were seeded in serum-free RPMI and migrated toward media specific for stem cells (SCM) containing DMEM/F12 with human supplementation of 10 ng/mL bFGF, 20 ng/mL EGF and 5 μg/mL insulin along with 0.4% BSA (each from Sigma, St. Louis, MO). Routine invasion assays were performed for 24 hours and then stained with the Diffi-Quick Staining kit (Dade Behring, Deerfield, IL). Three to five microscopic fields (20×) were photographed and counted for each sample. Percent invasion was calculated as average number of cells/field (Matrigel) divided by average number of cells/field (control insert). Values were averaged from 2-5 independent experiments. For the isolation of cells from top 'non-invading' and bottom 'invading' cells, parallel invasion chambers were setup. For non-invading cells, the bottom of the membrane was scrubbed with a cotton swab and cells on top were harvested using 500 μL of Accutase (eBioscience, San Diego, CA) incubated at 37°C for 5 minutes. To obtain the invading cells, the top of the membrane was scrubbed with a cotton swab and the chambers were placed into another 24- well plate containing 500 μL of Accutase incubated at 37°C for 5 minutes.

### MeDIP Arrays

Matrigel invasion assays were carried out as previously described. For the isolation of DNA from both non-invasive and invasive cells the DNeasy kit from Qiagen (Valencia, CA) was used and parallel invasion chambers were setup. For non-invading cells, the bottom of the membrane was scrubbed with a cotton swab and cells on top were trypsinized and harvested in 200 μL of PBS followed by the direct addition of lysis buffer or stored at -80°C. For bottom 'invading cells' the top of the membrane was scrubbed with a cotton swab and the membrane was removed and placed directly into lysis buffer or stored at -80°C until needed. A modified version of Agilent's (Santa Clara, CA) protocol for Mammalian ChIP on ChIP was used to capture methylated DNA with immunoprecipitation (MeDIP). DNA was quantified and 2 μg (or total yield if less) was digested with MseI overnight at 37°C. Linkers (JW102-5'-GCGGTGACCCGG-GAGATCTGAATTC-3' and JW103-5'-TAGAATTCAGATC-3') were ligated at 16°C using T4 ligase overnight and the next day used as input for the MethylCollector (Active Motif, Carlsbard, CA) assay to isolate methylated and non-methylated fractions of DNA. The kit utilizes histidine-tagged MeBP2 (methyl-binding protein 2) and magnetic bead separation. The isolated methylated and non-methylated DNA from each sample (as little as 5 ng) was then amplified in a series of PCR reactions following the mammalian ChIP on ChIP protocol. The input DNA was labeled with Cy3-dUTP and the methylated DNA with Cy5-dUTP and then immediately applied to Agilent's 2 × 244 K Human Promoter Tiling Arrays for 40 hours at 65°C. The arrays were scanned using a Gene Pix 4000B scanner (Molecular Devices, Sunnyvale, CA) with GenePix Pro software version 6.1 and extracted using Agilent's Feature Extraction software version 9.5.3.1. The data was annotated using Agilent's ChIP Analytics software version 4.0. Normalization was carried out using a blank subtraction model and statistical stringency (p-value) between 0.01-0.05 was applied using a Whitehead Per-Array Neighbourhood Analysis. This analysis allowed for the determination of differentially methylated genes between non-invasive and invasive cells. Ingenuity core analysis was carried out to determine which pathways are of functional significance based on the gene lists identified http://www.ingenuity.com/. Genomatix software was used to determine transcription factor binding sites (matrix). A perfect match to the matrix gets a score of 1.00 (each sequence position corresponds to the highest conserved nucleotide at that position in the matrix), a "good" match to the matrix usually has a similarity of >0.80.

Mismatches in highly conserved positions of the matrix decrease the matrix similarity more than mismatches in less conserved regions.

### Methylation Specific polymerase chain reaction (MSP-PCR)

A total of 1 μg of DNA extracted from total (parental) DU145 and LNCaP cells was bisulfite modified using the EpiTect Bisulfite kit from Qiagen. PCR was performed using Platinum Taq Polymerase (Invitrogen) and 200 ng of either genomic or bisulfite treated DNA. The PCR method utilized was 94°C for 2 minutes, then 35 cycles (94°C for 30 seconds, 55°C for 30 seconds and 72°C for 1 minute) with a final extension of 10 minutes at 72°C. The unmethylated primers however were run with an annealing temperature of 42°C since their melting temperature values were drastically different from their methylated counter part. A portion of the PCR product was run on a 1% agarose gel containing ethidum bromide.

### Methylated primers

hBMX-Forward 5'- TGGTGAGACATCATGTGTTCCATT-3';

hBMX-Reverse 5'- ATGCCCTCAGTTGAGAACCACTGT-3';

hSOX1-Forward 5'-ATGATCAGCATGTACTTGCCCGC-3';

hSOX1- Reverse 5'-TCCGCTTCCTCCGTAGGTGATAAA-3'

### Unmethylated primers

hBMX-Forward 5'- TGGTGAGATATTATGTGTTTTATT-3';

hBMX-Reverse 5'- ATGTTTTTAGTTGAGAATTATTGT-3';

hSOX1-Forward 5'-ATGATTAGTATGTATTTGTTTGT-3';

hSOX1-Reverse 5'-TTTGTTTTTTTTGTAGGTGATAAA-3'

### Quantitative real time polymerase chain reaction (QRT-PCR)

Total RNA was isolated using TRIzol (Invitrogen Corporation, Carlsbad, CA). RNA from 'top' cells was isolated using a cell pellet acquired from trypsinizing cells from one membrane after bottom cells were removed with a cotton swab. Conversely, RNA from the bottom cells was isolated by combining three membranes where the top cells were removed using a cotton swab. The membranes were pooled and placed in TRIzol for 10 minutes at room temperature, and the conventional procedure for isolation of RNA was then followed. To increase the yield of RNA, 5 μg of linear acrylamide (Ambion, Austin, TX) was added prior to precipitation of RNA with isopropanol. Additionally to increase overall yield, 100 ng of RNA was amplified using the MessageAmp aRNA Amplification Kit (Ambion, Austin, TX). cDNA was prepared using the SuperScript^®^III First-Strand Synthesis System (Invitrogen Corporation, Carlsbad, CA). Quantitative real time polymerase chain reaction (qRT-PCR) analysis was performed using a StepOne Real-time PCR machine (Applied Biosystems, Foster City, CA) with TaqMan Gene Expression Assay reagents and probes (Applied Biosystems). A total of 4 μL of cDNA was used in a 20 μL reaction resulting in a 1:5 dilution. The following FAM labeld human probes were used: BMX (Hs00174139_m1), IRX3 (Hs00273561_s1), SOX1 (Hs01023894_m1), MCL-1 (Hs00172036_m1), MYC (Hs00153408_m1), STAT3 (Hs01047580_m1), SURVIVIN (Hs00977611_g1) and 18S rRNA (Hs99999901_s1). Relative fold induction of mRNA was compared between non-invasive and invasive cells using the Delta-Delta CT method of quantitation, and 18S rRNA was used as a loading control.

### shRNA of *Bmx *and *Sox1*

The Trans-Lentiviral pTRIPZ system from Open Biosystems (Huntsville, AL) was used to introduce shRNA against BMX (Clone ID: V2THS_150067) and SOX1 (V2THS_197330) along with a non-silencing control vector. The vectors were transfected into HEK239T cells which were seeded in serum-free media at 60% confluency in 10 cm^2 ^dishes using the Arrest-In reagent provided in the kit. The cells were transfected for 6 hours and then replaced with complete media. After 24 and 48 hours lentiviral supernatants were harvested, spun at 1500 rpms, and filtered using a 0.45 μM filter to clear them. The viral titer was mixed 1:1 with DU145 media and placed on sub-confluent DU145 cells for 4-6 hours and changed to complete media. The next day media containing 1 μg/mL of doxycycline (Sigma) was added to ensure efficient transfection/infection has occurred. Efficient transfection was observed using a TET inducible TurboRFP (red fluorescent protein) upstream of the shRNA that appears red upon successful infection. The cells were selected for 2 weeks in 1 μg/mL of puromycin (Sigma). Single cell clones were then generated and lowered expression was confirmed using Western blotting.

### Western Blotting and sub-cellular fractions

Total cell lysates were prepared using RIPA buffer (Sigma) and sub-cellular fractions using the NE-PER Nuclear Protein Extraction Kit (Thermo Scientific, Rockford,IL). Samples were loaded onto a 4-20% Tris-glycine gel and transferred to a PVDF membrane. The membranes were blocked at room temperature for 45 minutes in 5% non-fat milk in TBS-Tween (0.05%). Primary antibodies were as follows: BMX (Abcam-59360), pBMX (Cell Signaling-3211S), STAT3 (Santa Cruz-SC482), pSTAT3/Tyr705 (Cell Signaling-9131S), SOX1 (Cell Signaling 4194S) and Actin (Abcam-8227-50) and incubated overnight at 4°C. The membrane was washed 3× for 10 minutes each using TBS-T (0.1%). Secondary antibody was applied for 1 hour at room temperature (infrared goat-anti rabbit or mouse in the 800 channel) and washed. The membrane was developed using the Odyssey from Licor (Lincoln, NE). Protein loading was normalized using actin as a control. Densitometry analysis was performed using ImageJ (NIH, Bethesda, MD).

### Proliferation Assays

Cells were seeded overnight in a 96 well plate in 100 μL of regular media at a density of 2000 cells per well. Cell proliferation was measured using the CellTiter-Glo assay from Promega on Day 1, 3, 5 and 7 using 100 μL of reagent and an incubation time of 20 minutes. The relative luciferase units (RLU) were quantified using a Tecan Infinite 200 plate reader.

### Prostatosphere Formation Assays

LNCaP and DU145 cells were seeded at 1000 cells per mL in replacement media SCM supplemented with KO Serum Replacement (Invitrogen) for LNCaP or B27 (Invitrogen) for DU145 cells in non-adherent 6 well plates coated with Hydrogel (Corning Life Sciences, Chemlsford, MA). The prostatospheres were generated for 5-7 days and then quantified or RNA extracted.

### Immunofluorescence

Staining of invasive or non-invasive cells was performed directly on the Matrigel membrane. Duplicate invasion chambers were used for each antibody; one each for staining invasive cells or non-invasive cells. Cells not being stained were removed from each insert, and cells of interest were fixed to the membrane in 4% para-formaldehyde for 15 minutes at 25°C and permeabilized with 0.5% saponin in PBS for 15 minutes at 25°C followed by a series of washes with PBS. Non-specific antibody binding sites were blocked for 15 minutes with 1% BSA in PBS containing 0.1% Tween-20 (PBS-T). Cells were incubated with either anti-pBMX antibody in PBS-T, SOX1 (Cell Signaling, Danvers, MA), or pSTAT3 (Millipore/Upstate Technologies, Billerica, MA (4°C, overnight). Following 3× PBS-T washes, infrared goat anti-rabbit Alexa 488 (Molecular Probes, Carlsbad, CA) was added for 1 hour at 25°C using a 1:500 dilution in PBS-T and again washed, then air-dried. Membranes were mounted on glass slides with Vectashield containing DAPI (Vector Laboratories, Burlingame, CA). Cells were visualized with a Zeiss-510 L5 confocal microscope where separate images were obtained for Alexa-488 and DAPI fluorescence, as well as overlays and 10 slice Z-stacks. Images were analyzed using the Zeiss LSM5 Image Browser (version 3.2.0.115) and further prepared in Adobe Photoshop CS. "Non-invasive" cells were stained on the topside of the membrane, while "invasive cells" were stained on the underside of the membrane. Controls using the secondary antibody and no primary antibody indicated that little, if any, fluorescence was contributed by non-specific binding of this antibody (data not shown).

### Immunoprecipitation

Protein was extracted using RIPA buffer (Sigma) and lysates were incubated with either SOX1, STAT3 or BMX (same antibodies used in Western Blotting) overnight at 4°C with rotation. The next day Protein A sepharose beads were added to the lysate and incubated for 3 hours with rotation at 4°C. The lysate was then spun at 13,000 rpms in a benchtop centrifuge and washed 3× with RIPA buffer. Before loading on a 4-20% Tris-Glycine SDS-Page gel (Invitrogen) 2× loading buffer was added and upon completion the gel was transferred to a PVDF membrane. The membrane was blocked for 45 minutes using 5% non-fat milk in TBS-T (0.1%). The membrane was then incubated overnight at 4°C using either primary antibodies SOX1 or STAT3 diluted in blocking buffer to confirm a direction interaction. The membrane was washed 3× for 10 minutes each using TBS-T (0.1%). Secondary antibody was applied for 1 hour at room temperature (infrared goat-anti-rabbit in the 800 channel) and washed. The membrane was developed using the Odyssey from Licor. Protein loading was normalized using actin from pervious Westerns.

### EMSA

The Licor EMSA buffer kit was used according to the manufacturer's instructions. Infrared (IR) and unlabeled STAT3 oligos were ordered from IDT and used at 0.625 fmoles/reaction.

Wildtype probes (WT): (800-IR channel and unlabeled)

F- 5'-GATCCTTCTGGGAATTCCTAGATC-3';

R- 5'-GATCTAGGAATTCCCAGAAGGATC-3'

Mutant probes (MU): (700-IR channel)

F- 5'-GATCCTTCTGGGCCGTCCTAGATC-3';

R- 5'-GATCTAGGACGGCCCAGAAGGATC-3';

Mutant oligos and unlabled wildtype oligos were used at 200-fold molar excess. A total of 20 μg of nuclear protein extract was incubated with 1× binding buffer (100 mM Tris, 500 mM KCl, 10 mM DTT; pH 7.5), Poly (dl·dC) 1 μg/μL (in 10 mM Tris, 1 mM EDTA, pH 7.5), 25 mM DTT/2.5% Tween-20, 1% NP-40, 100 mM MgCl_2_, and 50% glycerol for 20 minutes at room temperature shielded from light. For supershift experiments, extracts were pre-incubated with 5 μg of STAT3 antibody at 4°C for 30 minutes. DNA/protein complexes were visualized on a native 6% Tris-Borate-EDTA polyacrylamide gel. Gels were immediately removed from cassettes and scanned using the Odyssey in both the 700 and 800 channels.

### Meta-analysis on patient databases

Oncomine and Gene Expression Omnibus (GEO) databases were queried to identify associations between genes. GEO database is available at http://www.pubmed.org (GEO profiles) and provides raw expression data from several gene expression arrays. Oncomine 4.2 database analysis tool is available with a subscription at http://www.oncomine.org. Selected data was compared for gene expression levels in prostate primary tumor samples as well as their respective metastatic specimens. Data have been selected from [17] because this study was an integrated molecular profiling of gene expression in prostate cancer samples. In this work, a significant concordance between expression of *Sox1 *and *Stat3 *mRNA was found to correlate with the aggressiveness of the sample.

### Statistical Analysis

All statistical calculations were performed using GraphPad Prism Version 5. Comparisons between groups were carried out using either a Student's pair-wise t-test, or a One or Two-way ANOVA with a Bonferroni post-test wherever each test was applicable. Error bars represent the Standard Error of the Mean (SEM) and each experiment has been completed at least twice with samples in triplicate.

## Results

### Identification of differentially methylated genes in invasive sub-populations of cells

**Figure 1:**
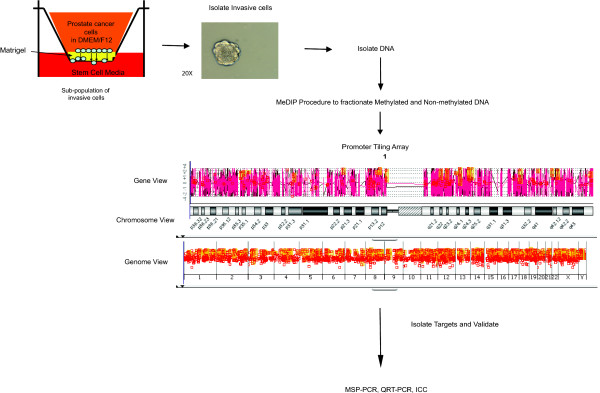
**Isolation and MeDIP analysis of invasive prostate cancer cells**. Matrigel-coated 24-well inserts (8 μM pore size) and non-coated control inserts purchased from BD Biosciences were used according to manufacturer's instructions. A range of 70,000-100,000 cells was seeded for the invasion. Cells were seeded in serum-free RPMI and migrated toward media specific for stem cells (termed SCM). For the isolation of DNA from top "non-invading" and bottom "invading" cells, parallel invasion chambers were set up. For non-invading cells, the bottom of the membrane was scrubbed with a cotton swab, and cells on top were harvested using 500 μL of trypsin incubated at 37°C for 5 minutes. To obtain the invading cells, the top of the membrane was scrubbed with a cotton swab, and the membranes were placed at -80°C until DNA extraction was performed or the cells were harvested with trypsin, as previously mentioned. DNA was extracted using a DNeasy kit (Qiagen), where the cell-based protocol was used to isolate DNA for the top cells, while the tissue extraction method was used to isolate the invaded cells from the previously stored membrane. Total genomic DNA was digested with MseI overnight, and methylated DNA was then collected using MethylCollector kit (Active Motif). Input and methylated DNA was then amplified with 2 rounds of PCR and labeled with either Cy5 for methylated or Cy3 for total DNA. The samples were combined and applied to Agilent's Human Promoter Tiling Array for 40 hours at 65°C. The arrays were then scanned with an Axon scanner (4000B) using GenePixPro version 6.1. The data were extracted using Agilent's Feature Extraction software 9.3.5.1, and differences in promoter methylation of genes in non-invasive and invasive cells were compared using Agilent's ChIP Analytics software version 4.0. Selected targets were then verified for methylation status using methylation-specific PCR, qRT-PCR and ICC.

**Figure 2:**
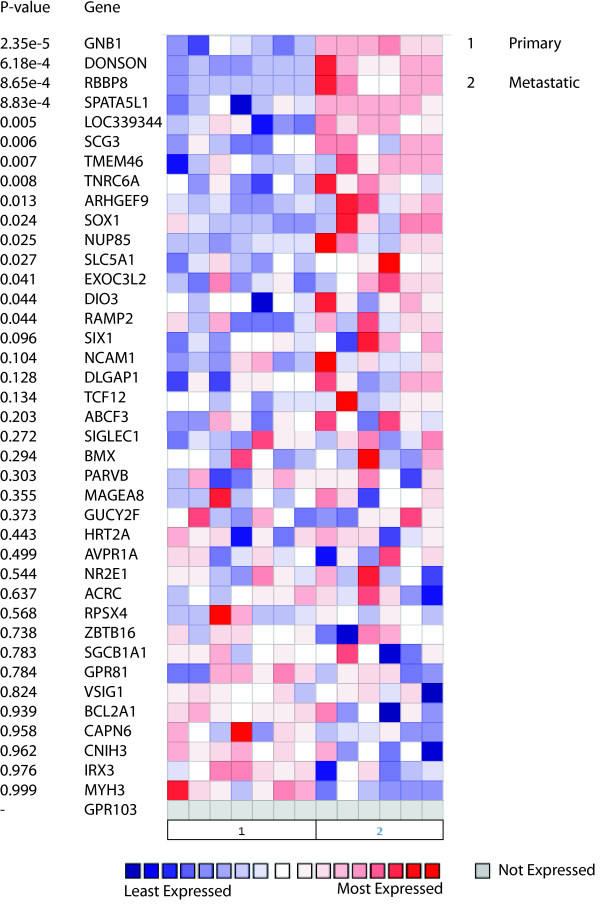
**Oncomine analysis of overlapping targets methylated in both LNCaP and DU145 cells**. Isolated targets from the methylation arrays overlapping in LNCaP and DU145 cells were analyzed in Oncomine 4.2 (Ann Arbor, MI). The heat map represents raw data from the Varambally over-expression in prostate cancer analysis comparing primary tissue and metastatic tissue [17]. Expression is in terms of normalized over-expression units. The P-value represents a student's t-test comparing primary and metastatic expression and the gene ID is provided. Genes of interest included *Sox1 *(p = 0.024) since it has high homology to the stem cell gene *Sox2 *and albeit demonstrating significance, *Bmx *(p = 0.294), since it has previously been implicated in prostate cancer regulation.

Individual promoter tiling arrays were performed to analyze global CpG promoter methylation for both non-invasive and invasive cell isolates from both LNCaP and DU145 (Figure 1). The cells were allowed to invade the Matrigel toward a highly defined media called stem cell media (SCM) [18]. It was then determined which genes were methylated in the non-invasive cells and not in the invasive fraction of cells. This analysis determined that 869 probes were differentially methylated in the non-invasive LNCaP fraction compared with the invasive and 1015 for DU145 (Additional File 1, Tables S1A and S1B). A very small subset of 44 overlapping genes was methylated in the non-invasive cells and not in the invasive population from both of the prostate cancer lines analyzed. These included genes involved in development such as *Irx3, Six1 *and *Sox1*, as well as a type-III 5" deiodinase (*Dio3*), and an embryonic version of myosin (*Myh3*) (Table 1). Using the Oncomine database we investigated changes in expression patterns for these methylated targets, and we found a significant association between progression of prostate cancer and metastasis with expression of a number of genes including G protein, beta-1 subunit (*Gnb1*), retinoblastoma binding protein 8 (*rbbp8*), secretogranin III (*Scg3*) and *Sox1 *(Figure 2). Albeit a number of these proteins have been shown to play a role in cancer, we chose to investigate the role of *Sox1 *in our model since it is very homologous to the induced pluripotent stem cell (iPS) regulator *Sox2*, and has been shown to play a role in progression of lung and nasopharyngeal cancer [19,20]. We also chose to investigate bone marrow tyrosine kinase gene in chromosome X protein (*Bmx*) since it has been shown to regulate hematopoiesis [21] and play a role in the regulation of prostate cancer [22]. However, from our Oncomine analysis *Bmx *was not shown to significantly affect prostate cancer metastasis (Figure 2).

**Table 1:** Summary of genes methylated within the non-invasive LNCaP and DU145 cell lines

Gene	Agilent ID	Location
ABCF3	A_17_P02824570	chr3:185384633-185384688
ACRC	A_17_P11784482	chrX:70712439-70712498
ARHGEF9	A_17_P11762981	chrX:62889218-62889272
AVPR1A	A_17_P08402446	chr12:61828587-61828646
BCL2A1	A_17_P09781583	chr15:78049283-78049342
BMX	A_17_P11607973	chrX:15428990-15429037
CAPN6	A_17_P11887436	chrX:110391469-110391527
CNIH3	A_17_P00830574	chr1:222866130-222866189
DIO3	A_17_P09520078	chr14:101092190-101092234
DLGAP1	A_17_P10514555	chr18:3832861-3832920
DONSON	A_17_P11359769	chr21:33880442-33880501
EXOC3L2	A_17_P17147769	chr19:50426839-50426891
GNB1	A_17_P00002239	chr1:1744644-1744703
GPR103	A_17_P03395862	chr4:122518949-122518996
GPR81	A_17_P08659974	chr12:121778515-121778572
GUCY2F	A_17_P11879791	chrX:108613896-108613952
HRK	A_17_P08637968	chr12:115801241-115801285
hsa-mir-346	A_17_P07370831	chr10:88012234-88012278
hsa-mir-507	A_17_P12010468	chrX:146120196-146120255
hsa-mir-542	A_17_P11969030	chrX:133503290-133503349
HTR2A	A_17_P08830482	chr13:46365855-46365914
IRX3	A_17_P10024825	chr16:52875157-52875205
LOC339344	A_17_P10976156	chr19:51085104-51085163
MAGEA8	A_17_P12020625	chrX:148768376-148768435
MYH3	A_17_P10227610	chr17:10497672-10497716
NCAM1	A_17_P08036168	chr11:112332051-112332110
NR2E1	A_17_P04970834	chr6:108588654-108588713
NUP85	A_17_P10465401	chr17:70708211-70708270
PARVB	A_17_P11521863	chr22:42723842-42723888
RAMP2	A_17_P10328303	chr17:38161563-38161609
RBBP8	A_17_P10568640	chr18:18762400-18762459
RPS4X	A_17_P11786429	chrX:71410283-71410342
SCG3	A_17_P09654436	chr15:49755368-49755423
SCGB1A1	A_17_P07831562	chr11:61937609-61937653
SIGLEC1	A_17_P11026333	chr20:3615793-3615841
SIX1	A_17_P16707873	chr14:60183180-60183224
SLC5A1	A_17_P11476138	chr22:30766279-30766324
SOX1	A_17_P09141969	chr13:111764575-111764619
SPATA5L1	A_17_P09626282	chr15:43477779-43477838
TCF12	A_17_P09676944	chr15:54992977-54993036
TMEM46	A_17_P08732705	chr13:25520762-25520811
TNRC6A	A_17_P09967235	chr16:24643725-24643784
VSIG1	A_17_P11874787	chrX:107171900-107171959
ZBTB16	A_17_P08041891	chr11:113430193-113430252

**Genes methylated within the non-invasive population of LNCaP and DU145 cell lines**. Once extracted from the Chip Analytics software, a list of genes which were methylated in the non-invasive cells, yet not in the invasive population was generated. The Agilent ID corresponds to the probe location on the chromosome

### Verification of methylation array data

**Figure 3:**
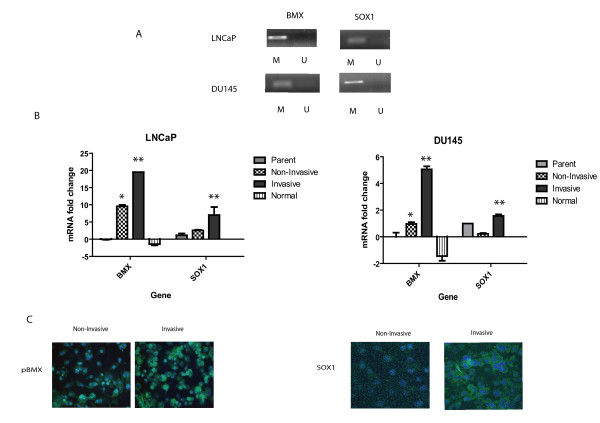
**Validation of methylated targets in LNcaP and DU145 cells**. **A) **DNA was extracted using the DNeasy kit and total of 1 μg from parental (total) LNCaP and DU145 cells was bisulfite modified using the EpiTect Bisulfite kit from Qiagen. MS-PCR was performed using Platinum Taq Polymerase (Invitrogen) and 200 ng of either genomic of bisulfite treated DNA was used. The samples were visualized using a 1% agarose gel and ethidium bromide. Both *Sox1 *and *Bmx *are methylated in the LNCaP and DU145 cell lines. **B) **Total RNA was isolated using TRIzol and qRT-PCR analysis was performed using a StepOne Real-time PCR machine with TaqMan Gene Expression Assay reagents and probes. Isolation of DNA and cDNA from non-invasive and invasive cells was carried out as previously described in materials and methods. Relative fold induction of mRNA was compared between non-invasive and invasive cells using the Delta-Delta CT method of quantitation where the parental lines were set at 1.0 as the control, and 18S rRNA was used as a loading control. Increased levels of both *Sox1 *and *Bmx *are seen in invasive LNCaP and DU145 cells compared to the non-invasive and parental lines. Normal human prostate RNA was used as a control. A Two-way ANOVA with a Bonferroni post-test was performed to compare groups and * represents a p-value of < 0.05 comparing parental to non-invasive cells and ** comparing parental to invasive cells. **C) **Staining of invasive or non-invasive cells was performed directly on the Matrigel membrane. Cells were incubated with either anti-pBMX antibody or SOX1 overnight and goat anti-rabbit Alexa-488 was added for 1 hour. Membranes were mounted on glass slides with Vectashield containing DAPI and visualized with a Zeiss-510 L5 confocal microscope. Images were analyzed using the Zeiss LSM5 Image Browser (20×) and further prepared in Adobe Photoshop CS. Increased levels of pBMX and SOX1 are seen in invasive cells compared to the non-invasive cells on top of the membrane.

To verify the results from our methylation specific promoter tiling arrays, we performed methylation specific PCR (MS-PCR) where primers were designed around the probe sequences identified from the arrays. Both *Bmx *and *Sox1 *were found to be methylated in the parental (total) LNCaP and DU145 cell lines (Figure 3A), representing the non-invasive phenotype. To determine if this pattern of methylation correlated with the level of gene expression, real time quantitative PCR (qRT-PCR) was performed. Significant differences in the expression of *Bmx *and *Sox1 *were seen when comparing the expression in non-invasive and invasive cell populations in both LNCaP and DU145 cell lines (Figure 3B) (Two-way ANOVA; *compares non-invasive to parental and ** compares invasive to parental, p < 0.05). To further validate the results, immunocytochemistry (ICC) was performed to analyze differences in protein expression between non-invasive and invasive cells. There is significantly higher expression of activated BMX and SOX1 in the invasive versus non-invasive cells (Figure 3C). Therefore, we validated the methylation and resultant decreased expression of BMX and SOX1 in the non-invasive cells.

### Functional role of Bmx and Sox1 during invasion

**Figure 4:**
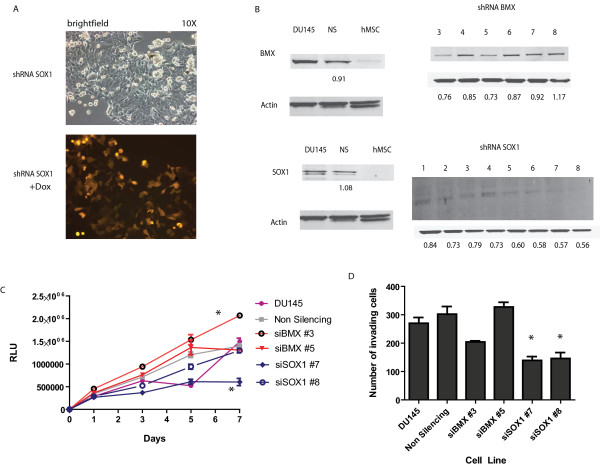
**Functional Role of SOX1 during invasion**. **A) **The Trans-Lentiviral pTRIPZ system from Open Biosystems was used to introduce shRNA against BMX, SOX1 or a non-silencing control vector in DU145 cells. The cells were selected for 2 weeks in 1 μg/mL of puromycin and single cell clones were generated. To induce expression of the shRNA 1 μg/mL of doxycycline was added. The plasmid is designed to have a TET inducible TurboRFP upstream of the shRNA and they should appear red upon successful infection. **B) **Lowered expression was confirmed using Western blotting. Follow up experiments were conducted using BMX clone 3 and 5 and SOX1 clone 7 and 8 since they demonstrated the most significant decrease in protein expression. Fold changes represent samples normalized to actin and the control level of expression. **C) **Proliferation assays were conduced using Cell Titer-Glo kit and assayed on Day 1, 3, 5 and 7. More proliferation is indicated by an increase in relative luciferase units (RLUs). *denotes statistical significant p < 0.05 compared to vector transfected cells. A significant decrease was observed in shSOX1 #7 cells compared to vector transfected cells, and a significant increase was observed in shBMX #5 cell line. **D) **Matrigel invasion assays were conducted for 24 hours toward SCM. Top cells were removed and bottom cells were stained with the Diff-Quick staining kit from Dade Behring. Cells were counted using 4 independent fields per sample and 2 chambers were used per cell line. *denotes statistical significance p < 0.05 compared to vector transfected cells. Both shSOX #7 and #8 demonstrated significant decreases in invasion toward SCM compared to vector transfected cells.

To further determine the role of *Bmx and Sox1 *during the process of invasion we performed the invasion assay with DU145 cells stably infected with shRNAs directed against *Sox1*or *Bmx *(Figure 4). A significant decrease in expression of SOX1 and BMX following induction with 1 μg/mL of doxycycline (Dox) for 24 hours was first verified using western blotting. Upon induction with Dox, the shRNA is turned on and a downstream red fluorescent protein (RFP) demonstrates efficiency of this induction (Figure 4A). Densitometry analysis was performed to compare expression of individual clones with the NS cells, and no significant differences in protein expression were seen using the non-silencing (NS) controls (Figure 4B). In addition, SOX1 shRNA cells demonstrated a significant decrease in proliferation compared to either the parental cell line (total cells) or the NS infected line (Figure 4C), as well as a significant decrease in invasion toward SCM (Figure 4D) (p-value < 0.05). However, there was not a significant difference using the shBMX lines, except for a slight reduction in invasion using clone #3. Interestingly, a small increase in proliferation was seen with the shBMX clones (Figure 4C). Further promoter tiling array analysis using two short term cultures primary prostate tumor cell lines, PCSC1 and PCSC2, determined that *Sox1*, and not *Bmx*, was methylated in the invasive population of cells (Additional File 2, Table S2A and B). Overall, we demonstrate that *Sox1*is differentially methylated within the invasive CSC population and the shRNA studies indicate it could be selectively targeted to block invasion.

### Role of SOX1 during differentiation

In addition to the method presented here, prostate TICs (tumor initiating cells) can also be isolated by culturing total cells in SCM where structures called prostatospheres are generated [18,23-25]. The prostatospheres are multicellular globes that develop from cells that survive anchorage-independent conditions *in vitro*, and are frequently used when analyzing the ability of TICs to self-renew or differentiate upon the addition of serum. Using this assay as a model, a greater number of prostatospheres were isolated from DU145 NS cells compared to shSOX1 cells (Additional File 3, Figure S1A and B). When invasive DU145 cells were isolated and cultured in SCM, prostatospheres were maintained for up to 3 passages (Additional File 3, Figure S1C) and if these cells were further cultured in the presence of 1% human serum (Additional File 3, Figure S1C), the vector control (NS) cells rapidly differentiated and proliferated, while the shSOX1 cells did not (Additional File 3, Figure S1D). These observations suggest that not only does *Sox1 *play a role in regulating invasion, but it can also regulate the maintenance of 'stem-ness' in culture.

### Ingenuity pathway analysis defines pathways of differentially methylated genes within invasive sub-populations of cells

Each data set of differentially methylated genes was then extracted and uploaded to the Ingenuity server to identify common gene pathways that are regulated during the process of invasion. The most conserved functional pathways between the cell lines are cellular development, cell growth and proliferation, as well as organismal development, nervous system development and function, and tissue development (Additional File 4, Table S3). The full list from the Ingenuity pathway analysis is also included (Additional File 5, Figure S2A and S2B). Additionally, the IL-6 signaling pathway involving STAT3 had a significant number of contributing methylated genes, a pathway recently found to play a significant role in cancer stem cell regulation [26-34] (Additional File 6, Figure S3A).

### Inhibitor studies further determine the role of IL-6/STAT3 pathway in invasion

**Figure 5:**
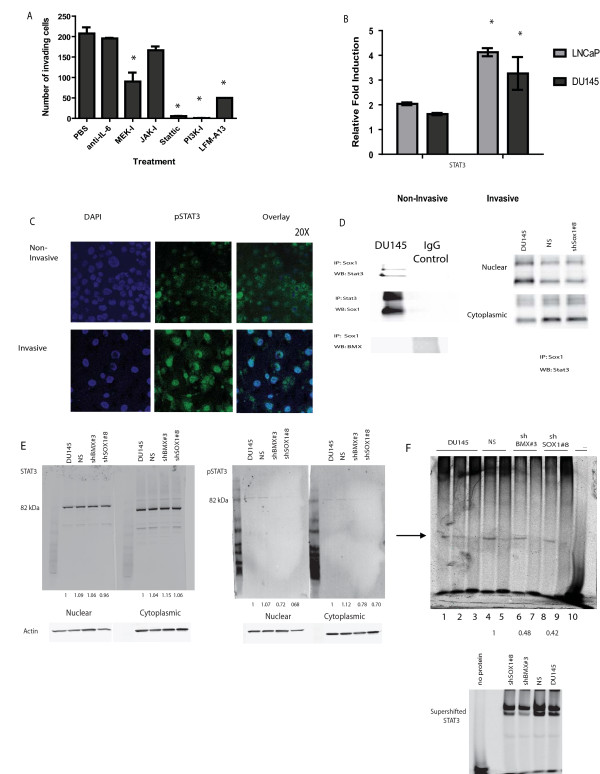
**Direct interaction between SOX1 and STAT3**. **A) **Matrigel invasion assays were performed for 24 hours toward SCM using DU145 cells in the presence of the anti-IL-6, the PI3K inhibitor LY294002, a small molecular inhibitor of MEK called U0126 (thus downstream inhibition of extracellular-related kinase (ERK1 and ERK2) mediated responses), a small molecule inhibitor JAK called AG490 (Janus Kinase) and an inhibitor of its partner signal transducers and activators of transcription-3 (STAT3) called Static or the Tec kinase family inhibitor LFM-A13.Significant differences were observed between control cells and those cells treated with U0126, Stattic, LY294002 and LFM-A13. **B) **qRT-PCR analysis was performed as mentioned in Figure 3. *denotes statistical significant p < 0.05 compared to the non-invasive cells. Increased levels of *Stat3 *are seen invasive LNCaP and DU145 cells compared to the parental lines. **C) **Staining of pSTAT3 in invasive or non-invasive DU145 cells was performed directly on the Matrigel membrane and carried out as previously described in Figure 3. **D) **DU145 lysates were incubated with either SOX1, STAT3 or BMX overnight at 4°C with rotation. Samples were then incubated with Protein A-agarose beads to isolate complexes. Membranes were then incubated overnight at 4°C using primary antibodies for STAT3 or SOX1. The membrane was developed using the Odyssey from Licor. Protein loading was normalized using actin as a control. **E) **Western blotting for STAT3 and pSTAT3 in sub-cellular protein extracts from DU145, NS, shBMX#3 or shSOX1#8. **F) **STAT3 EMSA: Each lane contains WT-IR STAT3 oligos. Lane 1-3 DU145, 4 and 5 NS, 6 and 7 shBMX#3, 8 and 9 shSOX1 #8 and 10 contains no protein. Lane 2 contains excess MU-IR STAT3 and lanes 3, 5, 7 and 9 contain excess unlabeled WT probe. Supershifited samples appear below and only contain WT-IR STAT3.

Based on the information generated from Ingenuity, we chose to determine how the IL-6 pathway might be regulating this process of invasion. A number of inhibitors of downstream targets of IL-6 regulation were tested for their ability to block invasion toward SCM. We included a neutralizing antibody to interleukin-6 (IL-6) to test what effect this may have upstream. Downstream of the receptor, the following inhibitors were used; the PI3K inhibitor LY294002, small molecular inhibitor of MEK called U0126 (thus downstream inhibition of extracellular-related kinase (ERK1 and ERK2) mediated responses), a small molecule inhibitor of JAK (Janus Kinase) called AG490 and an inhibitor of its partner signal transducers and activators of transcription-3 (STAT3) called Stattic (Figure 5A). Additionally, we tested the ability of the Tec kinase family inhibitor LFM-A13 based on the potential involvement of BMX during invasion (Figure 5A). The inhibitors which demonstrated the greatest effect at blocking invasion included Stattic, LY294002, and LFM-A13 (Figure 5A). However, a proliferation assay determined that Stattic could be preventing invasion because it was either cytotoxic to the cells or causing them to undergo apoptosis (Additional File 6, Figure S3B). To eliminate this possibility, viable cells were isolated after treating the DU145 cell line with Stattic for 24 hours (data not shown). These cells, although viable as determined by trypan blue staining, were still unable to invade.

### Direct interaction between the differentially methylated SOX1 and STAT3

Since inhibition of STAT3 demonstrated such a profound effect on invasion toward SCM, we questioned its involvement with the epigenetically regulated targets. Although we did not observe methylation of *Stat3 *itself, in both cell lines, the mRNA expression of *Stat3 *was increased (p-value < 0.03) when comparing invasive cells to their non-invasive counterpart (Figure 5B). Protein expression of pSTAT3 was also found to be increased in the invasive cells (Figure 5C). Since both SOX1 and STAT3 are known to act as transcriptional activators after forming protein complexes with other proteins [35-40], and BMX is known to activate STAT3 itself [40], we determined whether STAT3 directly interacts with either SOX1 or BMX. An interaction between SOX1 and STAT3 was observed (Figure 5D), however not between STAT3 and BMX (Figure 5D). In addition, a significant decrease in the expression of activated pSTAT3 was seen in both sub-cellular fractions of the BMX and SOX1 shRNA infected cells (Figure 5E). However, there was no change in total expression of STAT3. Additionally, a significant decrease in STAT3 DNA binding activity was observed in both BMX and SOX1 shRNA infected cells (Figure 5F). Overall, we see an interaction between SOX1 and STAT3, and upon loss of either BMX1 or SOX1 expression we observe a loss of STAT3 activation.

**Figure 6:**
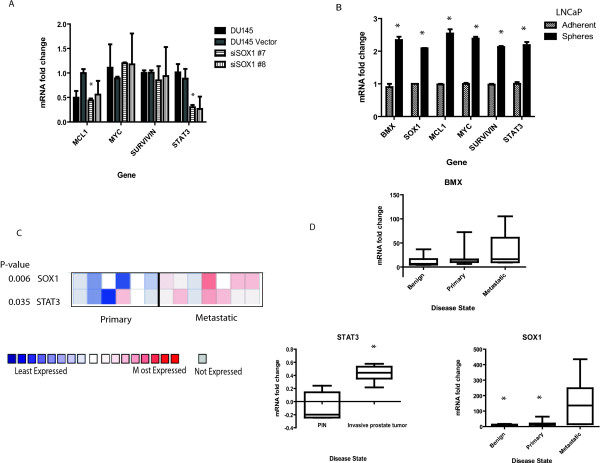
**Inhibitor studies further determine that the IL-6/STAT3 pathway is involved in invasion**. **A) **qRT-PCR demonstrating decreased expression of *Stat3 *in DU145 shSOX1 clone #7 cells and *Mcl-1*, a *Stat3 *target gene. No change was observed in *Myc *or *Survivin*. *denotes statistical significant p < 0.05 compared to the vector transfected line. **B) **Prostatospheres were generated by culturing LNCaP cells in SCM+KO for 7 days and qRT-PCR analysis was performed. Compared to adherent LNCaP cells, expression of *Bmx, Sox1 Mcl-1, Myc, Survivin*, and *Stat3 *was significantly increased in the stem-like prostatospheres. *denotes statistical significant p < 0.001 compared to the adherent cells. **C) **Correlation of *Sox1 *and *Stat3 *was analyzed in Oncomine 4.2 (Ann Arbor, MI). The heat map represents raw data from the Varambally over-expression in prostate cancer analysis comparing primary tissue and metastatic tissue. Expression is in terms of normalized over-expression units. The P-value represents a student's t-test comparing primary and metastatic expression. **D) **Using the GEO database, expression of *Sox1 *and *Bmx *were compared between benign, primary or metastatic prostate tissue and significant differences were observed in *Sox1*. For *Stat*3, a comparison between Prostate Intraepithelial Neoplasia (PIN) and invasive prostate tumor tissue yield a significant difference in expression. *denotes statistical significant p < 0.05 compared to either benign samples or PIN.

To further elucidate the connection between the SOX1 and STAT3, a decrease in the STAT3 target gene *Mcl-1 *and *Stat3 *itself were observed by qRT-PCR in shSOX1 clone #7 cells (Figure 6A). However, no change was observed for the STAT3 targets genes *Survivin *or *Myc *(Figure 6A). Finally, since prostatospheres are also a model for generating aggressive populations of cells in culture, we generated them from LNCaP cells and asked if STAT3 genes were affected. qRT-PCR analysis was performed and compared to adherent LNCaP cells, expression of *Stat3 *and *Stat3 *target genes *Mcl-1*, *Myc*, and *Survivin *were increased as well as *Bmx *and *Sox1 *(Figure 6B).

In order to determine what might be regulating the increased expression of *Stat3 *and *Sox1*, transcription factor binding sites were analyzed using Genomatix software. In both the *Stat3 *and *Sox1 *promoters there are a number of overlapping binding sites for transcription factors with a significant matrix value such as GATA-binding factors, RNA polymerase II transcription factor IIB (TFIIB), NeuroD/Beta2, TALE homeodomain class recognizing TG motifs, TCF11 transcription factor otherwise known as Nrf2, Nkx homeodomain factors, and finally the Zinc finger transcription factor RU49 also called Zipro1 (Additional File 7, Table S4). With this information, we can begin to understand why the methylation of *Sox1 *could serve as a master regulator of CSC invasion, thereby controlling its potential to undergo EMT and further metastasize.

Additional analysis using the GEO database determined that both *Sox1 *and *Stat3 *are expressed at higher levels in metastatic prostate cancer tissues and not *Bmx *(Figure 6C and 6D). Overall, we demonstrate that SOX1 is an epigenetically regulated target involved in the progression of prostate cancer, and is involved in signaling via the STAT3 pathway.

## Discussion

The process of epigenetic regulation by DNA methylation involves covalent modification of cytosine nucleotides at the C5 position in specific areas of CpG dinucleotides. The majority of methylated CpG dinucleotides are present in heterochromatic regions, and thus are unexpressed in the genome [41]. The process of methylation in mammals evolved as a method of silencing genes when their expression is not required. For example, the process of genomic imprinting involves DNA methylation where one allele of a gene, either maternal or paternal, is silenced [42]. This process only affects a few hundred genes within the genome, most of which encode for genes that regulate embryonic and neonatal growth [43]. Likewise, a number of CpG islands on one X chromosome are methylated during a process called X-chromosome inactivation [44]. This process ensures an equal amount of gene expression between males and females.

Using this model of invasion, we currently have developed a method to analyze differences in global CpG promoter methylation between total prostate cancer cells and their invasive population using promoter tiling arrays from Agilent. We identified a small subset of genes which were found to be differentially methylated between non-invasive and invasive LNCaP and DU145 cell lines. The results were highly intriguing because the majority of the genes normally function during human development (Additional File 4, Table S3). Based on previous data, these invasive cells demonstrated characteristics of true cancer stem cells (CSCs) [7]. It is becoming more evident that CSCs are not governed by the same type of genetic regulation as normal stem cells, and arguably may be an epithelial cell that has up-regulated pathways that have been previously observed in true stem cells. To determine the epigenetic profile of these invasive prostate cancer cells and putative TICs, we determined which genes are differentially methylated.

The appearance of *Sox1 *as one epigenetically regulated target presented the most interesting finding of this investigation. SOX proteins are transcription factors that are key regulators of determining neuronal cell fate, not only mammals, but also in *Drosophila*, *Xenopus*, and avian models [36]. Recently, much attention has been focused on these transcription factors since ectopic expression of *Sox2 *along with *Oct3/4*, *Klf4 *and *Myc *have been shown to reprogram murine fibroblasts to pluripotency, which in turn yields induced pluripotent stem (iPS) cells [45]. In our model, when expression of SOX1 was decreased in DU145 cells using shRNA, there was a significant reduction in invasion toward our stem cell media termed SCM (Figure 4D). Although SOX1 has yet to be implicated as a regulator of aggression in prostate cancer, it has been implicated as a marker of CSCs in breast cancer. Using either CD44^+^/CD24^- ^or CD133^+ ^cells isolated from *Brca1-deficient *mouse mammary tumors, expression of *Sox1 *was found to be significantly higher in these cells when compared to their counterparts [46]. In fact, expression of *Sox1 *was found to be 19.2-fold higher in CD44^+^/CD24^- ^compared to CD44^-^/CD24^+ ^cells, which represented the greatest change in any gene from this analysis [46].

The appearance of *Bmx *(also referred to as *Etk*) as a differentially methylated target was also interesting, yet not surprising, since this protein is a well-known regulator of prostate cancer. BMX is a family member of the Tec family of non-receptor tyrosine kinases that are predominately expressed in cells of hematopoietic origin, yet recently has also been shown to be expressed in arterial endothelium and a variety of epithelial cells [21,39,47,48]. Although BMX has a role in the formation of leukemia [21,49], our research is the first to demonstrate that BMX may play a significant role in the regulation of prostate cancer invasion and TICs. Although our shRNA studies against BMX did not demonstrate significant differences in invasion toward SCM, we were able to inhibit invasion of DU145 cells using the Tec family kinase inhibitor LFM-A13 without affecting normal cell proliferation (Additional File 6, Figure S3B), suggesting that this family of kinases may be indeed involved in metastasis.

After uploading our extensive list of differently methylated genes into the Ingenuity pathway analysis software, we observed that a number of the genes were members of the IL-6/STAT3 pathway. We tested a number of inhibitors of the IL-6 pathway for their ability to block invasion toward SCM. Small and non-significant effects of invasion were seen when inhibitors for MEK and JAK pathways, as well as a neutralizing antibody to IL-6 itself (Figure 5A). However, significant effects were seen using a PI3K inhibitor and a STAT3 inhibitor (Figure 5A). The role of PI3K signaling in prostate CSC regulation has been characterized, thus this observation is not too surprising [50]. The most pronounced effect, however, was observed with the STAT3 inhibitor Stattic. This drug inhibits binding of a phosphotyrosine-containing peptide derived from the gp130 receptor to the STAT3 SH2 domain with IC_50 _value of 5.1 ± 0.8 μM after 1 hr of incubation at 37°C [51]. The role of STAT3 in cancer progression has been known for sometime [52-56], and its role in CSC regulation has only recently been investigated. Higher levels of STAT3 have been demonstrated in CSCs isolated from liver, bone, cervical and brain cancers [26,27,29,57-59], and furthermore treatment of putative glioblastoma stem cells (GBM-SC) with Stattic results in a dramatic reduction in their formation [27]. Although the *Stat3 *gene itself was not methylated in any of our studies, qRT-PCR analysis demonstrated that compared to non-invasive cells, the invasive cells had a significant increase in expression of *Stat3 *(Figure 5B) and ICC detected an increase in active protein as well (Figure 5C). However, as seen in Figure S3B, there was a significant reduction in cell proliferation with Stattic treatment. To determine if this was the reason why we observed such a significant reduction in invasion, we took the remaining cells which survived treatment and further placed them through an invasion assay. The cells were unable to invade toward SCM, indicating that the cells resistant to Stattic-induced apoptosis were still sensitive at inhibiting invasion by lowering STAT3 (data not shown). A similar result was observed in the GBM-SCs, since different isolates of the cells responded differently to treatment with Stattic. The authors concluded that GBM-SCs derived in serum respond to Stattic by undergoing apoptosis, however in those derived using stem cell media they do not [27]. They state that this could be a result of certain GBM-SC lines being more differentiated, and are thus more sensitive to STAT3 inhibition.

Since inhibition of SOX1 with shRNA and BMX ultimately with LFM-A13 (data not shown, but LFM-A13 inhibited IL-6 mediated activation of BMX in LNCaP cells) decreased invasion toward SCM, we sought to determine if an interaction might be occurring between these differentially methylated genes and STAT3. To test this, an IP was performed to see if either BMX or SOX1 directly interact with STAT3. We found that only SOX1 could directly interact with STAT3 and not BMX (Figure 5D), and this interaction occurs in both the cytoplasm and the nucleus. In these sub-cellular fractions, we still see an association between SOX1 and STAT3 in shSOX1 cells since expression of the protein was not fully ablated (Figure 4B). Interestingly, decreased expression of either BMX or SOX1 does result in significantly less active STAT3 (Figure 5E) and a decrease in its DNA binding activity (Figure 5F). This observation is not too surprising since BMX has been shown to regulate such cellular processes as differentiation, motility, invasion, apoptosis, and more recently, when inhibited, a delay in tumor growth [22,60-66]. Specifically, within the prostate, BMX is up-regulated in tumors from both mouse and human specimens compared to benign tissues, and when over-expressed in cell lines, led to an increase in proliferation and elevated levels of AKT and STAT3 [22]. Albeit having a role in the formation of leukemia [21,49], our research is the first to demonstrate that BMX may play a significant role in the regulation of prostate CSCs.

Both STAT3 and SOX1 are transcription factors that regulate cell fate and differentiation; however a direct interaction between these proteins has never been identified. Future studies will be needed to determine what protein domains of each molecule are important for this interaction, as well as which promoters these transcription factors are regulating. However, the Oncomine and GEO data further support the observation that expression of both *Sox1 *and *Stat3 *are key genes regulating the progression of prostate cancer (Figure 6C and 6D). Regulation of *Sox1 *and *Stat3 *expression could occur coordinately since within their promoters they both contain transcription factor binding sites for NeuroD, TALE containing proteins, TCF11, and Nkxs (Additional File 7, Table S4). The TCF family of transcription factors regulates many patterns of development and activation of the TCF/LEF promoters. Recently, the Wnt proteins have been shown to regulate the 'stemness' of CSCs [67-70]. Additionally, expression of Nkx factors are required for neuronal cell fate, and interestingly, *Nkx*2.2, *Nkx*6.1 and *Irx3*, a NKX target, are also methylated in our study (Table 1) [71].

## Conclusions

Overall, our data demonstrates that *Sox1 *is methylated in two prostate cancer cell lines, LNCaP and DU145, and two short-term primary prostate cancer cultures, PCSC1 and PCSC2, yet not methylated in the invasive compartment of these cells. The expression of *Sox1 *was found to be correlated with increased levels of *Stat3 *in our invasive cells, and to directly interact with the protein product as well. Finally, both *Sox1 *and *Stat3 *were found to have increased expression in relation to the progression of prostate cancer in humans. Using our *in vitro *method to investigate invasion we can begin to understand which genes are epigenetically regulated in the invasive putative CSC population. The process of epigenetic regulation is complex, but we have begun to unravel it in these invasive cells from the prostate.

## Competing interests

The authors declare that they have no competing interests.

## Authors' contributions

LAM contributed to the conception and design, collection and/or assembly of data, data analysis and interpretation and manuscript writing. EMH contributed to the conception and design, data analysis and interpretation, final approval of manuscript and other (extensive editing). XZ contributed significantly to the collection and/or assembly of data. WLF contributed to the conception and design, financial support, final approval of manuscript and other (extensive editing).

All authors read and approved the final manuscript.

## Supplementary Material

Additional file 1 **Table S1: Total methylated gene lists generated by Chip Analytics software**. **A) **LNCaP **B) **DU145. Data represents those changes with a p-value ≤0.05 using nearest neighbor analysis. Genes are listed in alphabetical order according to their gene name. Click here for file

Additional file 2 **Table S2: Total methylated gene lists generated by Chip Analytics software**. **A) **PCSC1 **B) **PCSC2. Data represents those changes with a p-value ≤ 0.05 using nearest neighbor analysis. Genes are listed in alphabetical order according to their gene name. Click here for file

Additional file 3 **Figure S1: Prostatosphere formation and differentiation of DU145 and shSOX1 #7 cells**. DU145 cells were seeded 1000 cells per mL in replacement media SCM and supplemented with B27 in non-adherent 6 well plates coated with Hydrogel. The prostatospheres were generated for 5-7 days and then quantified. **A) **Comparison of DU145 spheres and those from clone #7 using the shRNA against SOX1. **B) **Number of spheres generated from DU145, NS and shSOX1 clone #7 cell lines. **C) **Ability of DU145 NS invasive cells to differentiate after addition of 1% human serum for 96 hours in culture and morphologically resemble DU145 NS cells. **D) **Comparison of differentiation potential of invasive cells isolated from DU145, NS and shSOX1 #7 cell lines. Click here for file

Additional file 4 **Table S3: Summary of significant functional gene pathways generated by Ingenuity software analysis for genes not methylated within the invasive cells**. Click here for file

Additional file 5 **Figure S2: Ingenuity analysis of methylation data demonstrating significant changes in functional gene pathways**. **A) **DU145 **B) **LNCaP. Click here for file

Additional file 6 **Figure S3**: **A) **Ingenuity analysis of methylation data demonstrating significant changes in canonical gene pathways in LNCaP. **B) **Proliferation assay for 24 hours using inhibitors from Figure 5A measured using CelTiter-Glo. Significant differences were seen between control cells and cells treated with Stattic. *denotes statistical significant p < 0.05 compared to the adherent cells. Click here for file

Additional file 7 **Table S4: Predicted transcription factor (TF) binding sites common to both the *sox1 *and *stat3 *promoters**. The potential TF factors sites were generated using the Genomatix software with full length promoter sequences for both genes. Click here for file

## References

[B1] Al-Hajj M Wicha MS Benito-Hernandez A Morrison SJ Clarke MF Prospective identification of tumorigenic breast cancer cells Proc Natl Acad Sci USA 2003 100 3983 3988 10.1073/pnas.0530291100 12629218 PMC153034

[B2] Graziano A d'Aquino R Tirino V Desiderio V Rossi A Pirozzi G The stem cell hypothesis in head and neck cancer J Cell Biochem 2008 103 408 412 10.1002/jcb.21436 17546610

[B3] Cariati M Purushotham AD Stem cells and breast cancer Histopathology 2008 52 99 107 10.1111/j.1365-2559.2007.02895.x 18171421

[B4] Kasper S Exploring the Origins of the Normal Prostate and Prostate Cancer Stem Cell Stem Cell Rev 2008 4 193 201 10.1007/s12015-008-9033-1 18563640 PMC11075662

[B5] Takaishi S Okumura T Wang TC Gastric cancer stem cells J Clin Oncol 2008 26 2876 2882 10.1200/JCO.2007.15.2603 18539967 PMC2743304

[B6] Lee CJ Dosch J Simeone DM Pancreatic cancer stem cells J Clin Oncol 2008 26 2806 2812 10.1200/JCO.2008.16.6702 18539958

[B7] Klarmann GJ Hurt EM Mathews LA Zhang X Duhagon MA Mistree T Thomas SB Farrar WL Invasive prostate cancer cells are tumor initiating cells that have a stem cell-like genomic signature Clin Exp Metastasis 2009 26 433 436 10.1007/s10585-009-9242-2 19221883 PMC2782741

[B8] Yu SC Bian XW Enrichment of cancer stem cells based on heterogeneity of invasiveness Stem Cell Rev 2009 5 66 71 10.1007/s12015-008-9047-8 19096941

[B9] Sarkar FH Li Y Wang Z Kong D Pancreatic cancer stem cells and EMT in drug resistance and metastasis Minerva Chir 2009 64 489 500 19859039 PMC2878773

[B10] Hollier BG Evans K Mani SA The epithelial-to-mesenchymal transition and cancer stem cells: a coalition against cancer therapies J Mammary Gland Biol Neoplasia 2009 14 29 43 10.1007/s10911-009-9110-3 19242781

[B11] Schmalhofer O Brabletz S Brabletz T E-cadherin, beta-catenin, and ZEB1 in malignant progression of cancer Cancer Metastasis Rev 2009 28 151 166 10.1007/s10555-008-9179-y 19153669

[B12] Morel AP Lievre M Thomas C Hinkal G Ansieau S Puisieux A Generation of breast cancer stem cells through epithelial-mesenchymal transition PLoS One 2008 3 e2888 10.1371/journal.pone.0002888 18682804 PMC2492808

[B13] Mani SA Guo W Liao MJ Eaton EN Ayyanan A Zhou AY Brooks M Reinhard F Zhang CC Shipitsin M The epithelial-mesenchymal transition generates cells with properties of stem cells Cell 2008 133 704 715 10.1016/j.cell.2008.03.027 18485877 PMC2728032

[B14] Bailey JM Singh PK Hollingsworth MA Cancer metastasis facilitated by developmental pathways: Sonic hedgehog, Notch, and bone morphogenic proteins J Cell Biochem 2007 102 829 839 10.1002/jcb.21509 17914743

[B15] Brabletz T Hlubek F Spaderna S Schmalhofer O Hiendlmeyer E Jung A Kirchner T Invasion and metastasis in colorectal cancer: epithelial-mesenchymal transition, mesenchymal-epithelial transition, stem cells and beta-catenin Cells Tissues Organs 2005 179 56 65 10.1159/000084509 15942193

[B16] Tsuji T Ibaragi S Shima K Hu MG Katsurano M Sasaki A Hu GF Epithelial-mesenchymal transition induced by growth suppressor p12CDK2-AP1 promotes tumor cell local invasion but suppresses distant colony growth Cancer Res 2008 68 10377 10386 10.1158/0008-5472.CAN-08-1444 19074907 PMC2605670

[B17] Varambally S Yu J Laxman B Rhodes DR Mehra R Tomlins SA Shah RB Chandran U Monzon FA Becich MJ Integrative genomic and proteomic analysis of prostate cancer reveals signatures of metastatic progression Cancer Cell 2005 8 393 406 10.1016/j.ccr.2005.10.001 16286247

[B18] Hurt EM Kawasaki BT Klarmann GJ Thomas SB Farrar WL CD44+ CD24(-) prostate cells are early cancer progenitor/stem cells that provide a model for patients with poor prognosis Br J Cancer 2008 98 756 765 10.1038/sj.bjc.6604242 18268494 PMC2259168

[B19] Huang DY Lin YT Jan PS Hwang YC Liang ST Peng Y Huang CY Wu HC Lin CT Transcription factor SOX-5 enhances nasopharyngeal carcinoma progression by down-regulating SPARC gene expression J Pathol 2008 214 445 455 10.1002/path.2299 18085523

[B20] Yamada Y Tahara M Miya T Satoh T Shirao K Shimada Y Ohtsu A Sasaki Y Tanigawara Y Phase I/II study of oxaliplatin with oral S-1 as first-line therapy for patients with metastatic colorectal cancer Br J Cancer 2008 98 1034 1038 10.1038/sj.bjc.6604271 18319719 PMC2275487

[B21] Kaukonen J Lahtinen I Laine S Alitalo K Palotie A BMX tyrosine kinase gene is expressed in granulocytes and myeloid leukaemias Br J Haematol 1996 94 455 460 8790141

[B22] Dai B Kim O Xie Y Guo Z Xu K Wang B Kong X Melamed J Chen H Bieberich CJ Tyrosine kinase Etk/BMX is up-regulated in human prostate cancer and its overexpression induces prostate intraepithelial neoplasia in mouse Cancer Res 2006 66 8058 8064 10.1158/0008-5472.CAN-06-1364 16912182

[B23] Garraway IP Sun W Tran CP Perner S Zhang B Goldstein AS Hahm SA Haider M Head CS Reiter RE Human prostate sphere-forming cells represent a subset of basal epithelial cells capable of glandular regeneration in vivo Prostate 70 491 501 19938015 10.1002/pros.21083 PMC2885946

[B24] Mulholland DJ Xin L Morim A Lawson D Witte O Wu H Lin-Sca-1+CD49fhigh stem/progenitors are tumor-initiating cells in the Pten-null prostate cancer model Cancer Res 2009 69 8555 8562 10.1158/0008-5472.CAN-08-4673 19887604 PMC2783355

[B25] Duhagon MA Hurt EM Sotelo-Silveira JR Zhang X Farrar WL Genomic profiling of tumor initiating prostatospheres BMC Genomics 11 324 10.1186/1471-2164-11-324 20500816 PMC2900264

[B26] Wang H Lathia JD Wu Q Wang J Li Z Heddleston JM Eyler CE Elderbroom J Gallagher J Schuschu J Targeting interleukin 6 signaling suppresses glioma stem cell survival and tumor growth Stem Cells 2009 27 2393 2404 10.1002/stem.188 19658188 PMC2825688

[B27] Sherry MM Reeves A Wu JK Cochran BH STAT3 is required for proliferation and maintenance of multipotency in glioblastoma stem cells Stem Cells 2009 27 2383 2392 10.1002/stem.185 19658181 PMC4391626

[B28] Gao H Priebe W Glod J Banerjee D Activation of signal transducers and activators of transcription 3 and focal adhesion kinase by stromal cell-derived factor 1 is required for migration of human mesenchymal stem cells in response to tumor cell-conditioned medium Stem Cells 2009 27 857 865 10.1002/stem.23 19350687

[B29] Tang Y Kitisin K Jogunoori W Li C Deng CX Mueller SC Ressom HW Rashid A He AR Mendelson JS Progenitor/stem cells give rise to liver cancer due to aberrant TGF-beta and IL-6 signaling Proc Natl Acad Sci USA 2008 105 2445 2450 10.1073/pnas.0705395105 18263735 PMC2268156

[B30] Daheron L Opitz SL Zaehres H Lensch MW Andrews PW Itskovitz-Eldor J Daley GQ LIF/STAT3 signaling fails to maintain self-renewal of human embryonic stem cells Stem Cells 2004 22 770 778 10.1634/stemcells.22-5-770 15342941

[B31] Nichane M Ren X Bellefroid EJ Self-regulation of Stat3 activity coordinates cell-cycle progression and neural crest specification Embo J 29 55 67 10.1038/emboj.2009.313 19851287 PMC2808363

[B32] Choi SC Kim SJ Choi JH Park CY Shim WJ Lim DS Fibroblast growth factor-2 and -4 promote the proliferation of bone marrow mesenchymal stem cells by the activation of the PI3K-Akt and ERK1/2 signaling pathways Stem Cells Dev 2008 17 725 736 10.1089/scd.2007.0230 18788932

[B33] Lawson DA Zong Y Memarzadeh S Xin L Huang J Witte ON Basal epithelial stem cells are efficient targets for prostate cancer initiation Proc Natl Acad Sci USA 107 2610 2615 10.1073/pnas.0913873107 20133806 PMC2823887

[B34] Garraway IP Sun W Tran CP Perner S Zhang B Goldstein AS Hahm SA Haider M Head CS Reiter RE Human prostate sphere-forming cells represent a subset of basal epithelial cells capable of glandular regeneration in vivo Prostate 2009 70 491 501 10.1002/pros.21083 PMC2885946 19938015

[B35] Seguin CA Draper JS Nagy A Rossant J Establishment of endoderm progenitors by SOX transcription factor expression in human embryonic stem cells Cell Stem Cell 2008 3 182 195 10.1016/j.stem.2008.06.018 18682240

[B36] Kan L Israsena N Zhang Z Hu M Zhao LR Jalali A Sahni V Kessler JA Sox1 acts through multiple independent pathways to promote neurogenesis Dev Biol 2004 269 580 594 10.1016/j.ydbio.2004.02.005 15110721

[B37] Gao SP Bromberg JF Touched and moved by STAT3 Sci STKE 2006 2006 pe30 10.1126/stke.3432006pe30 16835434

[B38] Gerhartz C Heesel B Sasse J Hemmann U Landgraf C Schneider-Mergener J Horn F Heinrich PC Graeve L Differential activation of acute phase response factor/STAT3 and STAT1 via the cytoplasmic domain of the interleukin 6 signal transducer gp130. I. Definition of a novel phosphotyrosine motif mediating STAT1 activation J Biol Chem 1996 271 12991 12998 10.1074/jbc.271.22.12999 8662591

[B39] Wen X Lin HH Shih HM Kung HJ Ann DK Kinase activation of the non-receptor tyrosine kinase Etk/BMX alone is sufficient to transactivate STAT-mediated gene expression in salivary and lung epithelial cells J Biol Chem 1999 274 38204 38210 10.1074/jbc.274.53.38204 10608894

[B40] Saharinen P Ekman N Sarvas K Parker P Alitalo K Silvennoinen O The Bmx tyrosine kinase induces activation of the Stat signaling pathway, which is specifically inhibited by protein kinase Cdelta Blood 1997 90 4341 4353 9373245

[B41] Jones PA Takai D The role of DNA methylation in mammalian epigenetics Science 2001 293 1068 1070 10.1126/science.1063852 11498573

[B42] Reik W Collick A Norris ML Barton SC Surani MA Genomic imprinting determines methylation of parental alleles in transgenic mice Nature 1987 328 248 251 10.1038/328248a0 3600805

[B43] Rugg-Gunn PJ Ferguson-Smith AC Pedersen RA Status of genomic imprinting in human embryonic stem cells as revealed by a large cohort of independently derived and maintained lines Hum Mol Genet 2007 16 Spec No. 2 R243 251 10.1093/hmg/ddm245 17911167

[B44] Wolf SF Jolly DJ Lunnen KD Friedmann T Migeon BR Methylation of the hypoxanthine phosphoribosyltransferase locus on the human X chromosome: implications for X-chromosome inactivation Proc Natl Acad Sci USA 1984 81 2806 2810 10.1073/pnas.81.9.2806 6585829 PMC345159

[B45] Park IH Zhao R West JA Yabuuchi A Huo H Ince TA Lerou PH Lensch MW Daley GQ Reprogramming of human somatic cells to pluripotency with defined factors Nature 2008 451 141 146 10.1038/nature06534 18157115

[B46] Wright MH Calcagno AM Salcido CD Carlson MD Ambudkar SV Varticovski L Brca1 breast tumors contain distinct CD44+/CD24- and CD133+ cells with cancer stem cell characteristics Breast Cancer Res 2008 10 R10 10.1186/bcr1855 18241344 PMC2374965

[B47] Schmidt U Boucheron N Unger B Ellmeier W The role of Tec family kinases in myeloid cells Int Arch Allergy Immunol 2004 134 65 78 10.1159/000078339 15133303

[B48] Ekman N Lymboussaki A Vastrik I Sarvas K Kaipainen A Alitalo K Bmx tyrosine kinase is specifically expressed in the endocardium and the endothelium of large arteries Circulation 1997 96 1729 1732 9323053 10.1161/01.cir.96.6.1729

[B49] Ekman N Arighi E Rajantie I Saharinen P Ristimaki A Silvennoinen O Alitalo K The Bmx tyrosine kinase is activated by IL-3 and G-CSF in a PI-3K dependent manner Oncogene 2000 19 4151 4158 10.1038/sj.onc.1203763 10962576

[B50] Dubrovska A Kim S Salamone RJ Walker JR Maira SM Garcia-Echeverria C Schultz PG Reddy VA The role of PTEN/Akt/PI3K signaling in the maintenance and viability of prostate cancer stem-like cell populations Proc Natl Acad Sci USA 2009 106 268 273 10.1073/pnas.0810956106 19116269 PMC2629188

[B51] Schust J Sperl B Hollis A Mayer TU Berg T Stattic: a small-molecule inhibitor of STAT3 activation and dimerization Chem Biol 2006 13 1235 1242 10.1016/j.chembiol.2006.09.018 17114005

[B52] Messina JL Yu H Riker AI Munster PN Jove RL Daud AI Activated stat-3 in melanoma Cancer Control 2008 15 196 201 18596671 10.1177/107327480801500302

[B53] Abdulghani J Gu L Dagvadorj A Lutz J Leiby B Bonuccelli G Lisanti MP Zellweger T Alanen K Mirtti T Stat3 promotes metastatic progression of prostate cancer Am J Pathol 2008 172 1717 1728 10.2353/ajpath.2008.071054 18483213 PMC2408430

[B54] Torres-Roca JF DeSilvio M Mora LB Khor LY Hammond E Ahmad N Jove R Forman J Lee RJ Sandler H Pollack A Activated STAT3 as a correlate of distant metastasis in prostate cancer: a secondary analysis of Radiation Therapy Oncology Group 86-10 Urology 2007 69 505 509 10.1016/j.urology.2006.11.006 17382154

[B55] Sanchez-Ceja SG Reyes-Maldonado E Vazquez-Manriquez ME Lopez-Luna JJ Belmont A Gutierrez-Castellanos S Differential expression of STAT5 and Bcl-xL, and high expression of Neu and STAT3 in non-small-cell lung carcinoma Lung Cancer 2006 54 163 168 10.1016/j.lungcan.2006.07.012 16959370

[B56] Wei D Le X Zheng L Wang L Frey JA Gao AC Peng Z Huang S Xiong HQ Abbruzzese JL Xie K Stat3 activation regulates the expression of vascular endothelial growth factor and human pancreatic cancer angiogenesis and metastasis Oncogene 2003 22 319 329 10.1038/sj.onc.1206122 12545153

[B57] Feng D Peng C Li C Zhou Y Li M Ling B Wei H Tian Z Identification and characterization of cancer stem-like cells from primary carcinoma of the cervix uteri Oncol Rep 2009 22 1129 1134 19787230 10.3892/or_00000545

[B58] Wilson H Huelsmeyer M Chun R Young KM Friedrichs K Argyle DJ Isolation and characterisation of cancer stem cells from canine osteosarcoma Vet J 2008 175 69 75 10.1016/j.tvjl.2007.07.025 17851099

[B59] Nilsson CL Dillon R Devakumar A Shi SD Greig M Rogers JC Krastins B Rosenblatt M Kilmer G Major M Quantitative phosphoproteomic analysis of the STAT3/IL-6/HIF1alpha signaling network: an initial study in GSC11 glioblastoma stem cells J Proteome Res 9 430 443 10.1021/pr9007927 19899826

[B60] Abassi YA Rehn M Ekman N Alitalo K Vuori K p130Cas Couples the tyrosine kinase Bmx/Etk with regulation of the actin cytoskeleton and cell migration J Biol Chem 2003 278 35636 35643 10.1074/jbc.M306438200 12832404

[B61] Busby JE Shih SJ Yang JC Kung HJ Evans CP Angiogenesis is not mediated by prostate cancer neuropeptides Angiogenesis 2003 6 289 293 10.1023/B:AGEN.0000029409.94626.64 15166497

[B62] He Y Luo Y Tang S Rajantie I Salven P Heil M Zhang R Luo D Li X Chi H Critical function of Bmx/Etk in ischemia-mediated arteriogenesis and angiogenesis J Clin Invest 2006 116 2344 2355 16932810 10.1172/JCI28123 PMC1551932

[B63] Pan S An P Zhang R He X Yin G Min W Etk/Bmx as a tumor necrosis factor receptor type 2-specific kinase: role in endothelial cell migration and angiogenesis Mol Cell Biol 2002 22 7512 7523 10.1128/MCB.22.21.7512-7523.2002 12370298 PMC135657

[B64] Rajantie I Ekman N Iljin K Arighi E Gunji Y Kaukonen J Palotie A Dewerchin M Carmeliet P Alitalo K Bmx tyrosine kinase has a redundant function downstream of angiopoietin and vascular endothelial growth factor receptors in arterial endothelium Mol Cell Biol 2001 21 4647 4655 10.1128/MCB.21.14.4647-4655.2001 11416142 PMC87133

[B65] Tu T Thotala D Geng L Hallahan DE Willey CD Bone marrow X kinase-mediated signal transduction in irradiated vascular endothelium Cancer Res 2008 68 2861 2869 10.1158/0008-5472.CAN-07-5743 18413754

[B66] Tsai YT Su YH Fang SS Huang TN Qiu Y Jou YS Shih HM Kung HJ Chen RH Etk, a Btk family tyrosine kinase, mediates cellular transformation by linking Src to STAT3 activation Mol Cell Biol 2000 20 2043 2054 10.1128/MCB.20.6.2043-2054.2000 10688651 PMC110821

[B67] Zeilstra J Joosten SP Dokter M Verwiel E Spaargaren M Pals ST Deletion of the WNT target and cancer stem cell marker CD44 in Apc(Min/+) mice attenuates intestinal tumorigenesis Cancer Res 2008 68 3655 3661 10.1158/0008-5472.CAN-07-2940 18483247

[B68] Katoh Y Katoh M FGF signaling inhibitor, SPRY4, is evolutionarily conserved target of WNT signaling pathway in progenitor cells Int J Mol Med 2006 17 529 532 16465403

[B69] Bisson I Prowse DM WNT signaling regulates self-renewal and differentiation of prostate cancer cells with stem cell characteristics Cell Res 2009 19 683 697 10.1038/cr.2009.43 19365403

[B70] Hurt EM Chan K Serrat MA Thomas SB Veenstra TD Farrar WL Identification of Vitronectin as an Extrinsic Inducer of Cancer Stem Cell Differentiation and Tumor Formation Stem Cells 2009 28 390 398 10.1002/stem.271 PMC3448441 19998373

[B71] Briscoe J Pierani A Jessell TM Ericson J A homeodomain protein code specifies progenitor cell identity and neuronal fate in the ventral neural tube Cell 2000 101 435 445 10.1016/S0092-8674(00)80853-3 10830170

