# Supplementary figures and images for: Epigenetic regulation of CpG promoter methylation in invasive prostate cancer cells
Source: Mol Cancer. 2010 Oct 7;9:267. doi: 10.1186/1476-4598-9-267 (PMC2958982; doi:10.1186/1476-4598-9-267)

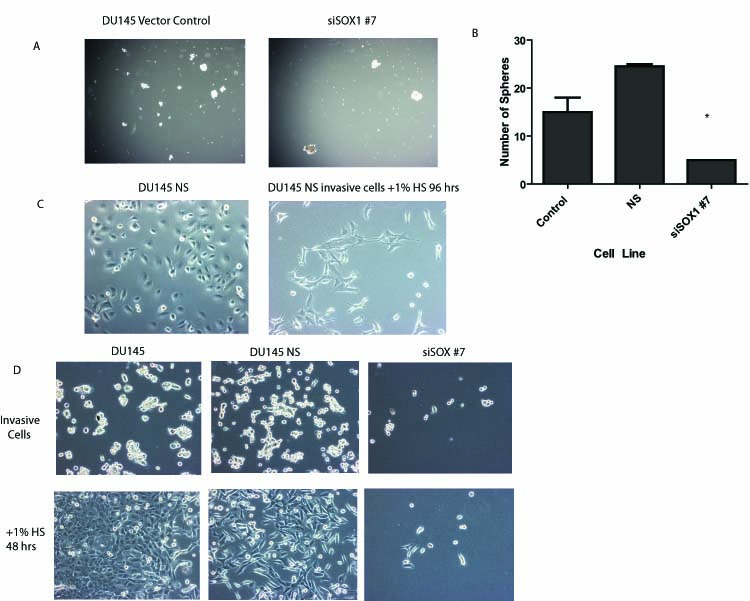

Supplement: Additional file 3 — Figure S1: Prostatosphere formation and differentiation of DU145 and shSOX1 #7 cells. DU145 cells were seeded 1000 cells per mL in replacement media SCM and supplemented with B27 in non-adherent 6 well plates coated with Hydrogel. The prostatospheres were generated for 5-7 days and then quantified. A) Comparison of DU145 spheres and those from clone #7 using the shRNA against SOX1. B) Number of spheres generated from DU145, NS and shSOX1 clone #7 cell lines. C) Ability of DU145 NS invasive cells to differentiate after addition of 1% human serum for 96 hours in culture and morphologically resemble DU145 NS cells. D) Comparison of differentiation potential of invasive cells isolated from DU145, NS and shSOX1 #7 cell lines. [file 1476-4598-9-267-S3.JPEG]
