# Supplementary material for: Epigenetic regulation of CpG promoter methylation in invasive prostate cancer cells
Source: Mol Cancer. 2010 Oct 7;9:267. doi: 10.1186/1476-4598-9-267 (PMC2958982; doi:10.1186/1476-4598-9-267)
Supplement: Additional file 4 — Table S3: Summary of significant functional gene pathways generated by Ingenuity software analysis for genes not methylated within the invasive cells. [file 1476-4598-9-267-S4.PDF]

Table S3: Genes methylated within the non-invasive population of LNCaP and DU145 cell lines

**Functions**	**LNCaP**	**DU145**
Behavior	X	
Cancer	X	
Cellular Assembly and Organization		X
Cell Development	X	X
Cell Function and Maintenance	X	
Cell Growth and Proliferation	X	X
Cell Morphology	X	
Cell-To-Cell Interaction	X	
Developmental Disorder		X
Embryonic Development		X
Genetic Disorder		X
Gene Expression	X	
Hematological System Development and Function		X
Organ Development		X
Organ Morphology		X
Organismal Development	X	X
Nervous System Development and Function	X	X
Neurological Disease	X	
Skeletal and Muscular System Development and Function		X
Tissue Development	X	X
Tumor Morphology	X	
Visual System Development and Function		X
