# Supplementary material for: Epigenetic regulation of CpG promoter methylation in invasive prostate cancer cells
Source: Mol Cancer. 2010 Oct 7;9:267. doi: 10.1186/1476-4598-9-267 (PMC2958982; doi:10.1186/1476-4598-9-267)
Supplement: Additional file 7 — Table S4: Predicted transcription factor (TF) binding sites common to both the sox1 and stat3 promoters. The potential TF factors sites were generated using the Genomatix software with full length promoter sequences for both genes. [file 1476-4598-9-267-S7.PDF]

Table S4: Transcription Factor binding sites are found in the promoters of both the *sox1* and the *stat3* promotors

**Detailed Family Information**	**Evidence**	**Matrix**
GATA binding factors	aagaGATTaccac	1
NeuroD, Beta2, HLH domain	gaagcacCTGCaa	1
NKX homeodomain factors	aatctTAATtgctggggaa	1
NKX homeodomain factors	ggcttcAAGTgtttttttt	1
RNA polymerase II transcription factor IIB	ccgCGCC	1
TALE homeodomain class recognizing TG motifs	acattcttGTCAaaagg	1
TCF11 transcription factor	GTCAttt	1
Zinc finger transcription factor RU49	aAGTAcc	1
